# *Mycoplasma genitalium* Protein of Adhesion Induces Inflammatory Cytokines via Cyclophilin A-CD147 Activating the ERK-NF-κB Pathway in Human Urothelial Cells

**DOI:** 10.3389/fimmu.2020.02052

**Published:** 2020-09-09

**Authors:** Lingling Li, Dan Luo, Yating Liao, Kailan Peng, Yanhua Zeng

**Affiliations:** ^1^Institute of Pathogenic Biology, Hengyang Medical College, University of South China, Hunan Provincial Key Laboratory for Special Pathogens Prevention and Control, Hengyang, China; ^2^Department of Dermatology and Venereology, The First Affiliated Hospital, University of South China, Hengyang, China

**Keywords:** *Mycoplasma genitalium*, CD147, cyclophilin A, inflammatory cytokines, NF-κB

## Abstract

*Mycoplasma genitalium* protein of adhesion (MgPa) plays an important role in the process of adhesion and invasion of host cells by *M. genitalium*, and is thus significant for its pathogenic mechanisms in host cells. Our previous study has demonstrated that cyclophilin A (CypA) is the receptor for MgPa in human urothelial cells (SV-HUC-1) and can, therefore, mediate the adherence and invasion of *M. genitalium* into host cells by interacting with MgPa. However, the specific pathogenesis of *M. genitalium* to host cells and the possible pathogenic mechanism involved in the interaction of MgPa and CypA have never been clarified. The study aimed to elucidate the mechanism involved in the pathogenicity of MgPa. Recombinant MgPa (rMgPa) induced extracellular CypA (eCypA) was detected in SV-HUC-1 cells by ELISA, and the interaction between CypA and CD147 was validated using co-localization and co-immunoprecipitation assay. In addition, both extracellular signal-regulated kinases (ERK) phosphorylation and NF-κB activation evoked by rMgPa-induced eCypA were also demonstrated. The findings of this study verified that rMgPa could induce the secretion of eCypA in SV-HUC-1 cells and thus promote the protein and mRNA expression of IL-1β, IL-6, TNF-α and MMP-9 via CypA-CD147 interaction and thus activating ERK-NF-κB pathway, which is beneficial to elucidate the pathogenesis and possible pathogenic mechanism of *M. genitalium* to host cells.

## Introduction

Sexually transmitted infections (STIs) have become a formidable threat to public health worldwide. Recently, *Mycoplasma genitalium* has been increasingly recognized as a sexually transmitted pathogen, but there is no defined public health response to this relatively newly identified STI so far (1). Moreover, besides being one of the causative agents responsible for non-gonococcal urethritis (NGU), *M. genitalium* is also related to diseases such as cervicitis, tubal infertility, pelvic inflammation and endometritis, which may catalyze the pathological effects of serious syndromes and permanent chronic conditions, such as infertility, chronic pelvic pain and ectopic pregnancy (2, 3). *M. genitalium* can cause opportunistic infections in AIDS patients by promoting the replication of the human immunodeficiency virus (HIV), thereby accelerate the death of patients (4, 5) and hence is also known as AIDS-related *Mycoplasma. M. genitalium* infection has been on the rise in recent years (6) warranting the need for effective therapeutic and prophylactic agents.

The binding of pathogens to membrane receptors located on host cells is critical for the adhesion and invasion of pathogens into host cells (7). Studies have verified that *M. genitalium* adheres to the surface of epithelial cells mainly through its peculiar apical structure and then invades into the cells, resulting in damage to the host cells via different mechanisms (8, 9). *M. genitalium* probably interacts with receptor proteins on the cells membrane surface via *M. genitalium* protein of adhesion (MgPa), invading into the cells and causing disease. *M. genitalium* is known to cause long-term infection in SV40-immortalized human urothelial cell line (SV-HUC-1) cells, resulting in the secretion of chronic inflammatory cytokines including interleukin-8 (IL-8), monocyte chemotactic protein 1 (MCP-1), and macrophage inflammatory protein 1β (MIP-1β) and promotion of responsiveness to secondary Toll-like receptor (TLR) stimulation (10). *M. genitalium* has also been reported to induce IL-6, IL-7, IL-8, MCP-1, and granulocyte-macrophage colony-stimulating factor (GM-CSF) secretion in a 3-dimensional epithelial cell model (11).

Cyclophilin A (CypA), first extracted from bovine thymus by Handschumacherl, is a highly conserved protein with peptidyl-prolyl *cis*-*trans* isomerase (PPIase) activity and is mainly distributed in the cytoplasm and in organelles such as nucleus, endoplasmic reticulum and mitochondria of all species (12, 13). It plays crucial roles in intracellular signal transduction, protein folding and transport, and modulation of transcriptional activity of other proteins. Extracellular CypA (eCypA), secreted by host cells during an infection by a pathogenic microorganism and oxidative stress can induce the expression of inflammatory cytokines and chemokines, which plays an important role in the process of inflammation (14, 15). Our previous study has shown that CypA present on the cell membrane acts as the potential receptor for MgPa in SV-HUC-1 cells (16). However, the specific pathogenesis and possible pathogenic mechanism mediated by the interaction between MgPa and CypA remains to be further understood.

Therefore, research such as the present study, which aimed at further elucidating the pathogenic mechanism involved in *M. genitalium* infection is required to ensure the development of effective targets for prophylactics and therapeutics. SV-HUC-1 cells were used in this study to detect recombinant MgPa (rMgPa) induced eCypA by ELISA and co-localization and co-immunoprecipitation (Co-IP) assays were used to validate the interaction between CypA and CD147. The findings of this study facilitated a better understanding of the pathogenesis of *M. genitalium* and led to an elucidation of the different mediators of the pathogenic mechanism resulting in the *M. genitalium* infection. Such clear understanding of the disease process can lay a strong foundation for the development of effective management and treatment options.

## Materials and Methods

### Cell Culture

The SV-HUC-1 was bought from American Type Culture Collection (ATCC, CRL-9520) and cultured in F-12K medium (Gibco, United States) containing 10% fetal bovine serum (FBS, Gibco, Australia) and 1% penicillin-streptomycin solution (Beyotime, CAS C0222, China) at 37°C constant temperature in a 5% CO_2_ humidity cell incubator.

### Detection of eCypA by ELISA

SV40-immortalized human urothelial cells (5 × 10^5^) were seeded into 24-well plates (Corning, United States) and incubated with 0, 10, 20, 30, 40, and 50 μg/mL rMgPa, respectively and the optimal concentration of rMgPa was then selected to stimulate the cells for 0, 8, 16, 24, 32, 40, and 48 h, respectively. About 100 ng/mL lipopolysaccharide (LPS) was used as a positive control. Each set comprised of three duplicate wells. The cell culture supernatants were collected to perform ELISA as described by Zhu (17). The eCypA was detected using human CypA ELISA kit (USABIO, *shanghai*, China) according to the specifications.

### Detection of Intracellular CypA and CD147 Expression by Western-Blotting

SV40-immortalized human urothelial cells were washed with cold PBS three times and were then lysed with ice-cold radio immunoprecipitation assay (RIPA) buffer (Beyotime, P0013B, China) supplemented with phenylmethane sulfonyl fluoride (PMSF) followed by centrifugation at 4°C for 15 min. The supernatants were harvested by centrifugation and the protein concentrations were measured using the bicinchoninic acid (BCA) assay (Beyotime, P0010, China). SDS-PAGE was used to fractionate 20 μg of boiled protein and transferred to a polyvinylidene fluoride (PVDF) membrane, which was blocked with 5% skimmed milk and then incubated with rabbit anti-CypA (1:1000, Abcam, ab41684, United Kingdom), mouse anti-CD147 (1:1000, Abcam, ab666, United Kingdom), and rabbit anti-GAPDH (1:2000, BOSTER, BA2913, China) antibodies respectively overnight at 4°C. The membranes were then washed with tris-bufferes saline with Tween-20 (TBST) six times and incubated with corresponding horse-radish peroxidase (HRP)-conjugated goat anti-rabbit IgG (1:1000, Proteintech, BL003A, United States) and HRP-conjugated goat anti-mouse IgG (1:1000, ComWin Biotech, CW0102, China) for 60 min at 37°C. The protein expression was visualized using the chemiluminescent imaging system (Syngene, United States) and quantified with the Image J analysis software.

### Indirect Immunofluorescence Assay

Cells were pre-incubated with diverse concentrations of rMgPa for varying periods of time. SV-HUC-1 cells were blocked with F-12K medium including 10% FBS after being washed three times with cold PBS and fixed with 4% paraformaldehyde for 30 min. The cells were incubated with rabbit anti-CypA antibody (1:100, Abcam, ab41684, United Kingdom) and mouse anti-CD147 antibody (1:100, Abcam, ab666, United Kingdom), respectively, at 4°C overnight. The cells were then washed 3 times with phosphate-buffered saline containing Tween-20 (PBST), and incubated with corresponding fluorescein isothiocyanate (FITC)-conjugated AffiniPure goat anti-rabbit IgG (H + L) (1:100, ComWin Biotech, CW0114S, China) and tetramethylrhodamine (TRITC)-conjugated AffiniPure goat anti-mouse IgG (H + L) (1:100, ComWin Biotech, CW0152S, China), and 4′,6-diamidino-2-phenylindole (DAPI) (Beyotime, C1002, China) for 1 h at room temperature. The fluorescence stained cells were washed 3 times and photographed using TE2000-S inverted microscope (Nikon, Japan).

### Co-localization Assay and Co-immunoprecipitation of CypA and CD147

The co-localization assay between CypA and CD147 in SV-HUC-1 cells was carried out as described by Deng (16). Briefly, the SV-HUC-1 cells were cultured on sterile glass coverslips in 24-well plates (Corning, United States) for 24 h at 37°C constant temperature in a 5% CO_2_ humidity cell incubator. The cells were then pre-treated with 20 μg/mL rMgPa for 32 h followed by washing with cold PBS. The washed cells were then fixed using 4% paraformaldehyde, washed with PBS and then the treated cells were blocked with medium containing 10% FBS at room temperature for 2 h. The cells were washed and incubated with rabbit anti-CyPA antibody (1:100, Abcam, ab41684, United Kingdom) and mouse anti-CD147 antibody (1:100, Abcam, ab666, United Kingdom) at room temperature for 2 h followed by incubation with FITC-conjugated AffiniPure goat anti-rabbit IgG (H + G) (1:100, ComWin Biotech, CW0114S, China), TRITC-conjugated AffiniPure goat anti-mouse IgG (H + L) (1:100, ComWin Biotech, CW0152S, China), and DAPI (Beyotime, C1002, China) at room temperature for 1 h. The cells were washed and photographed using TE2000-S inverted microscope (Nikon, Japan).

The interaction between CypA and CD147 was further confirmed by Co-IP as described by Ghosh (18). Briefly, 100 μg protein lysates (RIPA buffer) were incubated with 20 μL of 50% Protein A/G-Agarose beads and a 2.5 μL mouse anti-CypA antibody (Abcam, ab58144, United Kingdom) or mouse anti-CD147 antibody (Abcam, ab666, United Kingdom) or 2.5 μL anti-IgG antibody at 4°C overnight. The Protein A/G-Agarose beads were then washed with PBS to eliminate the unbound compound followed by centrifugation for 5 seconds at 4°C. The agarose magnetic beads and antigen–antibody complexes were suspended with PBS and then the immune complex was mixed with 5 × SDS sample buffer and boiled for 10 min, and then subjected to Western blotting.

### Quantitative Reverse Transcriptase Polymerase Chain Reaction

Total RNA was extracted using TRIzol (Beyotime, China) and then converted to cDNA using BeyoRT II First Strand cDNA synthesis kit (RNase H minus). Quantitative reverse transcriptase polymerase chain reaction (qRT-PCR) was performed using the SYBR Green mix (Qiagen, Shanghai, China) in the LightCycle 96 apparatus (Roche, Basel, Switzerland) (19). The primers used are shown in Table 1.

**TABLE 1:** Primer sequences used in this study.

Gene name	Forward primers (5′–3′)	Reverse primers (5′–3′)
IL-1	GCCCTAAACAGATGAAG TGCTC	GAACCAGCATCTTCCTCAG
IL-6	AGCCCTGAGAAAGGAGA CATGTA	GGAGTGGTATCCTCTGTGAAGTCT
TNF-α	TGGCCCAGGCAGTCAGA	GGTTTGCTACAACATGGGCTACA
MMP-9	TTGACAGCGACAAGAAGTGG	CCCTCAGTGAAGCGGTACAT
GAPDH	GCACCGTCAAGGCTGAGAAC	TGGGAAGACGCCAGTGGA

The PCR conditions were as follows: 95°C for 10 min, followed by 40 cycles of 95°C for 15 s, 72°C for 20 s, and 56°C for 15 s. Data were normalized by the level of GAPDH expression in each of the samples described above. Results were obtained in triplicates.

### ELISA

SV40-immortalized human urothelial cells (1 × 10^6^/each well) were seeded in a 6-wells plate and incubated with antibiotic-free medium for 48 h at 37°C with 5% CO_2_, and then transfected with CypA siRNA (Origene Technologies, SR303662) or CD147 siRNA (Origene Technologies, SR300476) using Lipofectamine2000 according to the instructions. siRNA duplexes, at a final concentration of 20 nM were transfected into the cells, which were then stimulated by 20 μg/mL rMgPa for 8, 16, 24, and 32 h, respectively. Three compound holes were set up per group. The cellular supernatants were then assembled and centrifuged at 3000 rpm for 5min. The expression of MMP-9 was determined using a human ELISA kit (Neobioscience, Shenzhen, China) while the IL-1β, IL-6, and TNF-α expressions were determined using the human ELISA kits (Thermo Fisher Scientific, United States) as per the manufacturer’s instructions.

### Detection of ERK-NF-κB Signaling Pathway Triggered by rMgPa Induced eCypA by Western Blotting

The phosphorylations of extracellular signal-regulated kinases (ERK) and inhibitor of nuclear factor kappa B (IκBα) which enhanced by rMgPa induced eCypA were detected by western blotting. Firstly, CypA-siRNA, CD147-siRNA group, ERK and NF-κB inhibitor preincubation group were setted up followed by treatment of cells with 20 μg/m rMgPa for 24 h. The total proteins were extracted, separated and then transferred onto the membrane. The membranes were blocked and then incubated with rabbit anti-ERK (1:1000, CUSABIO, CSB-PA002421, China), anti-phospho-ERK (1:1000, CUSABIO, CSB-PA000749, China), anti-p-IκBα (1:2000, Abcam, ab133462, United Kingdom), and mouse anti-IκBα (1:2000, Abcam, ab12134, United Kingdom) antibodies respectively overnight at 4°C. The transferred membranes were washed with TBST and incubated with HRP-conjugated goat anti-rabbit IgG (1:1000, Proteintech, BL003A, United States) or HRP-conjugated goat anti-mouse IgG antibody (1:1000, Beijing ComWin Biotech, China) at 37°C for 60 min. The proteins were visualized with a chemiluminescent imaging system (Syngene, United States). Meanwhile, the mRNA and protein levels of inflammatory cytokines were verified by qRT-PCR (see section “Quantitative Reverse Transcriptase Polymerase Chain Reaction”) and ELISA (see section “Quantitative Reverse Transcriptase Polymerase Chain Reaction”), respectively.

### Detection of NF-κB Activation by Indirect Immunofluorescence Assay and Western Blotting

Recombinant MgPa induced NF-κB activation was detected by Indirect immunofluorescence assay. Briefly, the cells were stimulated with medium, DMSO, rMgPa, and rMgPa combined with NF-κB inhibitor, respectively for 2 h. The cells were fixed immediately with 4% formaldehyde for 30 min after being washed with PBS. The cells were then penetrated with 0.2% Triton X-100 for 10 min and blocked with medium containing 10% FBS for 2 h. This was followed by incubated with rabbit anti-NF-κB p65 antibody (1:100) (Proteintech, 10745-1-AP, United States) overnight at 4°C and then with Cy3-conjugated anti-rabbit IgG antibody (1:100, Proteintech, SA00009-2, United States) followed by DAPI staining at 37°C for 1 h. The translocation of NF-κB was imaged by TE2000-S inverted microscope (Nikon, Japan).

The translocation of p65 was further verified by western blotting. Briefly, cells were treated with medium or rMgPa and the extracted cytoplasmic and nuclear proteins were collected as per manufacturer’s instructions. The protein concentrations were determined by BCA assay (Beyotime, China) based on which 20 μg protein was boiled, separated, and transferred to a PVDF membrane, which was blocked with 5% skimmed milk. The membranes were incubated with anti-p65 (1:1000, Proteintech, United States), anti-histone H3 (1:1000, CUSABIO, China), and anti-GAPDH (1:1000, CUSABIO, China) antibodies overnight at 4°C. The membranes were washed and incubated with HRP-conjugated goat anti-rabbit IgG (1:1000, Proteintech, United States) and HRP-conjugated goat anti-mouse IgG (1:1000, Beijing ComWin Biotech, China) respectively for 1 h at 37°C. The chemiluminescent imaging system (Syngene, United States) was then used to visualize the protein as described in a previously study (12).

### Statistical Analysis

All experiments were performed in triplicates and all data were expressed as the mean ± SD. The statistical evaluation was analyzed using the independent *t*-test for the comparison of two samples and using a one-way ANOVA test for comparisons of multiple samples. A *p* value less than 0.05 was considered to indicate a significant difference.

## Results

### rMgPa Induced eCypA Secretion in SV-HUC-1 Cells

**FIGURE 1:**
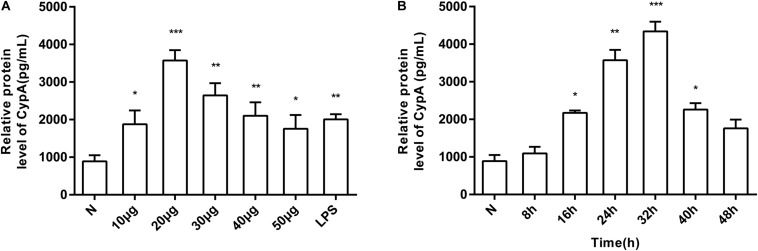
Induction of SV-HUC-1 cells by rMgPa to secrete eCypA in dose- and time-dependent manner. **(A)** SV-HUC-1 cells were stimulated with different concentrations of rMgPa for 24 h. The concentrations of eCypA were determined by ELISA. **(B)** SV-HUC-1 cells were stimulated by rMgPa (20 μg/mL) for the indicated time, the eCypA expressions were measured by ELISA. *p < 0.05, **p < 0.01, ***p < 0.001. N stands for the untreated group.

The induction of eCypA secretion in SV-HUC-1 cells by rMgPa was investigated by treating the cells with varying concentrations of rMgPa at different time points, and the supernatants were collected to measure the amount of secreted eCypA by ELISA. Different concentrations of rMgPa could stimulate cells to secrete eCypA and 20 μg/mL of rMgPa was found to be the optimal concentration for induction of eCypA secretion (Figure 1A). rMgPa was also found to induce the secretion of eCypA in a time-dependent manner, which was found to achieve a peak at 32 h (Figure 1B). These results demonstrated that rMgPa could induce eCypA secretion in SV-HUC-1 cells.

### rMgPa Can Enhance CypA and CD147 Expression in Cells

**FIGURE 2:**
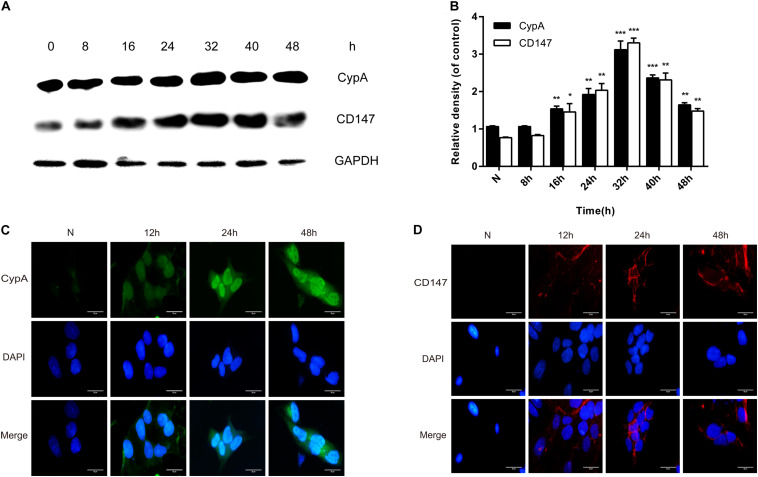
rMgPa-induced CypA and CD147 expression in SV-HUC-1 cells. **(A)** Western blot analysis of CypA and CD147 expressions in cells stimulated by rMgPa at different time points. **(B)** Values were expressed as fold change increases over unstimulated control (*n* = 3). *p < 0.05, **p < 0.01, ***p < 0.001. N stands for the untreated group. **(C)** Indirect immunofluorescence showed the increase of CypA in rMgPa-treated cells (10 × 100). **(D)** The expressions of CD147 in rMgPa-treated SV-HUC-1 cells were detected by indirect immunofluorescence (10 × 100), Scale bar, 50 μm.

Western blotting and indirect immunofluorescence were used to confirmthe enhancement of CypA and CD147 expressions in SV-HUC-1 cells byrMgPa. The expressions of CypA and CD147 were seen to increasegradually with the prolongation of rMgPa stimulation time, with the expressions being the highest when rMgPa stimulated the cells for 32 h as shown in Figures 2A,B. The results of indirect immunofluorescence demonstrated that the expressions of CypA (Figure 2C) located both in the cytoplasm and the cell membrane and CD147 (Figure 2D) located on the cell membrane were increased with the prolongation of stimulation time. These results validated that the expressions of CypA and CD147 could be promoted by rMgPa in SV-HUC-1 cells.

### Interaction Between CypA and CD147

**FIGURE 3:**
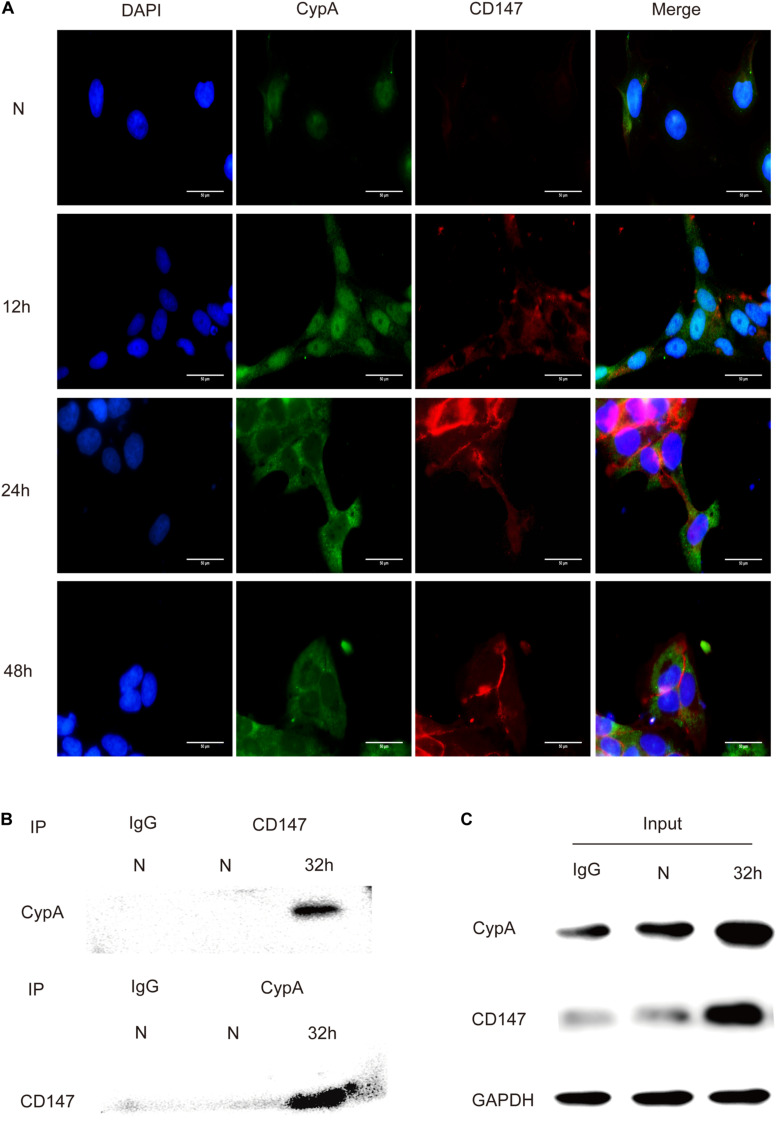
Detection of the interaction between CypA and CD147 by indirect immunofluorescence and co-immunoprecipitation. **(A)** The co-localization analysis of CypA and CD147 (10 × 100). Scale bar, 50 μm. **(B)** The co-immunoprecipitation of CypA with CD147 proved their interaction. **(C)** 5% of the reactions (100 μg) was used as input controls.

The *in vitro* interaction between CypA and CD147 was assessed using the co-localization and Co-IP assays. Results of the co-localization assay demonstrated that the expression levels of CypA and CD147 increased with the prolongation of stimulation time and that the co-localization of CypA and CD147 was found to be mainly at the cell membrane and partially in the cytoplasmic region (Figure 3A), which indicated that CypA and CD147 could interact in SV-HUC-1 cells. The results of the Co-IP tests validated that the CypA-CD147 complex was precipitated by CD147 antibody or CypA antibody, which indicated that CypA could directly interact with CD147 (Figure 3B). The input was as control (Figure 3C).

### rMgPa-Induced eCypA Can Trigger Secretion of Inflammatory Cytokines

**FIGURE 4:**
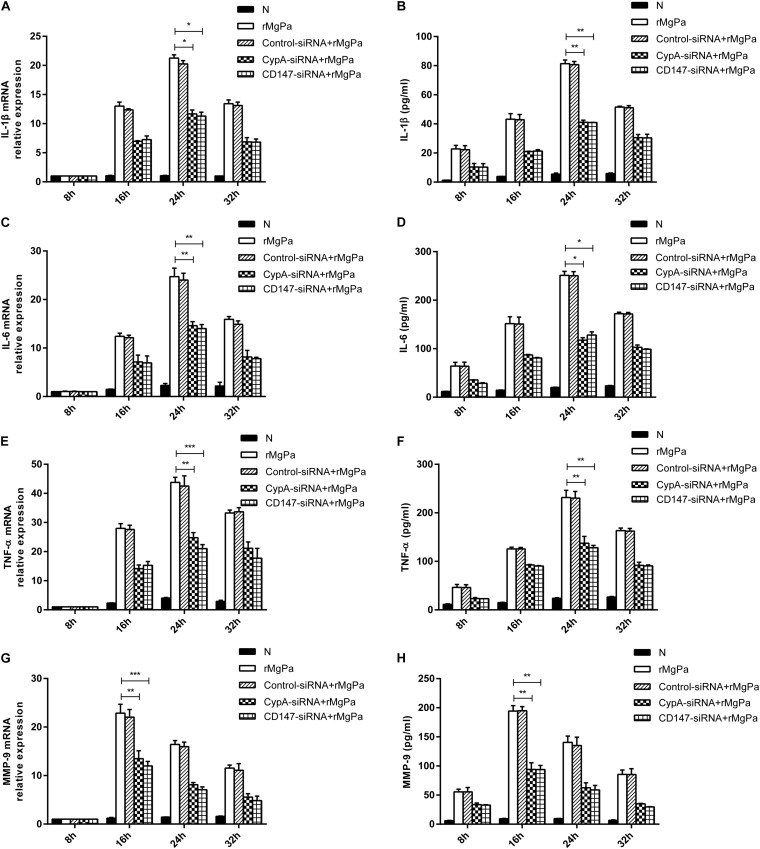
Inflammatory cytokines production triggered by rMgPa induced eCypA. The mRNA levels of IL-1β **(A)**, IL-6 **(C)**, TNF-α **(E)**, and MMP-9 **(G)** in SV-HUC-1 cells transfected with siRNA followed by stimulation with rMgPa measured at 8 h, 16 h, 24 h and 32 h by RT-qPCR after stimulation. Supernatants of protein levels of IL-1β **(B)**, IL-6 **(D)**, TNF-α **(F)**, and MMP-9 **(H)** were correspondingly collected to be measured by ELISA. ^∗^*p* < 0.05, ^∗∗^*p* < 0.01, ^∗∗∗^*p* < 0.001. N stands for the untreated group.

qRT-PCR and ELISA were used to confirmed the ability of rMgPa-induced eCypA to trigger the secretion of inflammatory cytokines in SV-HUC-1 cells. The mRNA levels of IL-1β (Figure 4A), IL-6 (Figure 4C), TNF-α (Figure 4E), and MMP-9 (Figure 4G) and the protein expression levels of IL-1β (Figure 4B), IL-6 (Figure 4D), TNF-α (Figure 4F), and MMP-9 (Figure 4H) were found to be significantly increased in rMgPa treated cells as compared to the control group. Treatment of SV-HUC-1 cells with 20 μg/mL rMgPa for 24 h resulted in the highest mRNA levels of IL-1β, IL-6, and TNF-α, with the mRNA level of MMP-9 being the highest at 16 h after rMgPa stimulation. In addition, the mediation of the rMgPa-induced inflammatory cytokines via CypA and CD147 was also evaluated. The mRNA expressions levels of these inflammatory cytokines at different time points for CypA-siRNA and CD147-siRNA interference groups were found to be substantially lower than the control siRNA group. These results demonstrated that CypA-siRNA and CD147-siRNA could negatively regulate rMgPa-triggered inflammatory cytokines. In summary, the above results verified that the production of rMgPa-triggered inflammatory cytokines was related to the eCypA secretion in SV-HUC-1 cells.

### rMgPa Induced eCypA Can Evoke the Secretion of Inflammatory Cytokines Mainly Through the ERK Signaling Pathway

**FIGURE 5:**
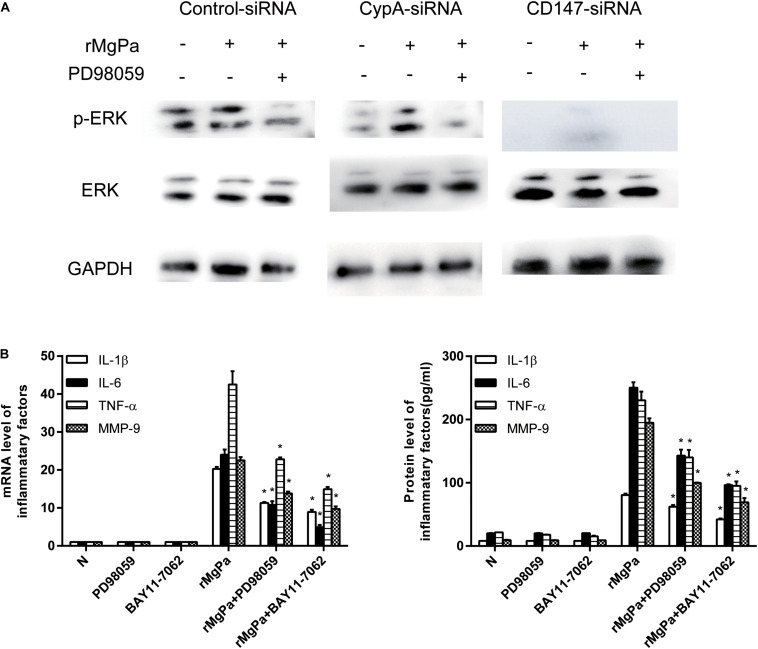
ERK signaling pathway mediated secretion of inflammatory cytokines evoked by rMgPa induced eCypA. **(A)** Western blot analysis of the cell lysates obtained from SV-HUC-1 cells transfected with siRNA and then stimulated with rMgPa, GAPDH was used as the control. **(B)** The mRNA levels of IL-1β, IL-6, TNF-α, and MMP-9 in the SV-HUC-1 cells preincubated with 30 μM PD98059 or BAY 11-7082 for 1 h before being stimulated with 20 μg/mL rMgPa were measured at 24 h by qRT-PCR. Supernatants were correspondingly collected after 24 h and measured by ELISA. *p < 0.05, N stands for the untreated group.

The ERK phosphorylation was detected by western blotting to demonstrate the ability of rMgPa induced eCypA to evoke the secretion of inflammatory cytokines via the ERK signaling pathway. The phosphorylation levels of ERK in the rMgPa-treated cells were found to be significantly increased as compared to the control group while the phosphorylation levels of ERK for the corresponding inhibitor pretreated cells were found to be significantly decreased (Figure 5A), which demonstrated that rMgPa could induce ERK phosphorylation. In addition, the phosphorylation levels of ERK for the cells transfected with the CypA-siRNA and CD147-siRNA were also found to be significantly decreased, indicating that rMgPa-induced phosphorylation of ERK was mediated via CypA and CD147 in SV-HUC-1 cells.

The results of qRT-PCR and ELISA assay showed that the ERK inhibitor had negative effects on mRNA transcription and cytokines secretion in rMgPa stimulated cells. The mRNA and protein expressions of IL-1β, IL-6, TNF-α and MMP-9 were also found to be significantly inhibited in the ERK inhibitor-pretreated cells as shown in Figure 5B. These results suggested that rMgPa induced eCypA could stimulate SV-HUC-1 cells to produce inflammatory cytokines by activating the ERK signaling pathway.

### rMgPa Can Trigger NF-κB Activation

**FIGURE 6:**
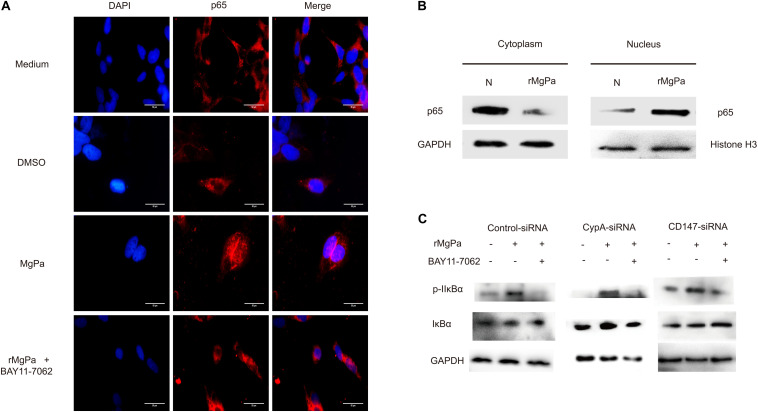
NF-κB activation triggered by rMgPa. **(A)** Indirect immunofluorescence based analysis of rMgPa induced nuclear translocation of p65 in SV-HUC-1 cells (10 × 100). The p65 nucleation phenomenon was obvious in the rMgPa-treated cells as compared to the medium or DMSO treated cells, and the translocation of p65 in the MgPa + NF-κB inhibitor pre-incubated cells was seen to be suppressed. Scale bars, 50 μm. **(B)** Western blotting analysis of the nuclear translocation of p65 in rMgPa-treated SV-HUC-1 cells. p65 existing in the cytoplasm of cells that were not stimulated by rMgPa, while in the cell nucleus after stimulation. **(C)** Western blotting analysis showed the increased phosphorylation of IκBα in SV-HUC-1 cells caused by NF-κB inhibitor BAY11-7082.

The mediation of the rMgPa-induced eCypA triggered inflammatory cytokines production via CypA-CD147-NF-κB pathway was confirmed by evaluating the effects of nuclear translocation of NF-κB p65 in rMgPa-treated cells by indirect immunofluorescence assay and western blotting. The p65 was found to be translocated from the cytoplasm to the nucleus in rMgPa treated cells and this p65 unclear translocation of p65 was found to be inhibited by NF-κB inhibitor (Figure 6A). The western blotting results also demonstrated that p65 was mainly expressed in the nucleus in rMgPa-stimulated cells, while it was found to be mainly present in the cytoplasm in unstimulated cells (Figure 6B).

The mediation of rMgPa induced NF-κB activation via CypA-CD147-ERK pathway, the was investigated by determining expression levels of IκBα and the phosphorylation levels of IκBα using western blotting. Results showed that the phosphorylation levels of IκBα for rMgPa-treated cells were significantly increased while the phosphorylation levels of IκBα for NF-κB inhibitor pre-incubated cells and CypA-siRNA and CD147-siRNA transfected cells were significantly decreased, as compared to the control group (Figure 6C). The effects of NF-κB inhibitor on mRNA transcription and cytokines secretion in rMgPa stimulated SV-HUC-1 cells were determined by qRT-PCR and ELISA assay. The mRNA and protein expressions of IL-1β, IL-6, TNF-α and MMP-9 were significantly inhibited in NF-κB inhibitor pretreated cells as compared to rMgPa treated cells (Figure 5B). These results validated that rMgPa activated NF-κB signaling pathway was mediated via CypA and CD147.

## Discussion

MgPa located at the apical part of *M. genitalium*, which is homologous to the adhesion protein P1 of *M. pneumonia*, which is the most widely distributed membrane protein and plays a crucial role in the pathogenesis of *M. genitalium*, such as adhesion, infection and inflammatory response (20). Our previous study validated that CypA, the potential receptor of MgPa, mediates the adherence and invasion of *M. genitalium* into urethral epithelial cells (16). However, the pathogenesis of *M. genitalium* and the possible pathogenic mechanism responsible for the interaction between CypA and MgPa has not been elucidated so far. In this study, it was verified that rMgPa could upregulate IL-1β, IL-6, TNF-α and MMP-9 expression through the secretion of eCypA, which interacts with CD147 and thus activates the ERK-NF-κB pathway in urethral epithelial cells.

The present study is the report, to the best of our knowledge, suggesting that CypA plays an important role in the pathogenesis of *M. genitalium* to host cells. CypA, widely existent in all kinds of organisms, is a kind of protein with a variety of biological functions such as protein folding, transportation, immunosuppression, viral infection, inflammation, and apoptosis (21). Moreover, CypA can also be secreted outside of cells to fight against inflammatory stimuli, hypoxia, infection, and oxidative stress (22). However, the mechanisms about how these factors regulate the eCypA secretion remain unclear so far. Jin ZG et al. found that the reactive oxygen species or oxidized low-density lipoprotein mediated eCypA secretion by vesicular secretory pathway (23). Su Z et al. indicated that autophage plays an important role in the exocytosis of CypA in rat aortic smooth muscle cells (RASMCs) (24). Although most eukaryotic secretory proteins are directed into the unconventional secretion (25). Unfortunately, the molecular mechanisms of eCypA secretion induced by rMgPa were not clarified in this study. Anyway, we will explore the possible mechanisms of rMgPa regulating eCypA secretion in SV-HUC-1 cells in our further studies. Extracellular CypA plays vital roles in acute and chronic inflammation-related human diseases such as viral infection, rheumatoid arthritis (26), periodontitis (27) and cardiovascular diseases, and CypA-1-specific inhibitors can be used as effective targets for treating inflammation-related diseases. A previous study has demonstrated that eCypA also has proinflammatory influences on endothelial cells by increasing their proliferation, migration, and invasion (28).

Previous studies have validated that extracellular matrix (ECM) metalloproteinase inducer (EMMPRIN/CD147) located on the cell membrane could serve as the main signaling receptor for CypA and thus mediate the chemotactic activity of cyclophilins toward immune cells (29, 30). CD4^+^ T cells activated by cyclophilin-mediated chemotaxis has been closely correlated with the growing expression of CD147, which play a key role in inflammatory responses (31). These findings demonstrated a correlation among CypA, CD147 expression and inflammatory responses. Moreover, studies have confirmed that the interaction of CypA and CD147 could cause MAPK activation (32). For example, Tsai found that hyperglycemia could induce HK-2 cells to secrete eCypA which in turn activated the p38 MAPK pathway (33). Moreover, Obchoei found that eCypA could interact with CD147 to activate the ERK1/2 and p38 MAPK signaling pathways and promote cell proliferation (34). Recent studies corroborated that eCypA could interact with CD147 and were thus internalized into cells thereby stimulating cells which could secrete inflammatory cytokines and chemokines, such as IL-8 and MCP-1 by activating NF-κB through the ERK1/2 pathway. Previous studies have proved that the proline 180 and glycine 181 residues in the extracellular domain of CD147 is the key amino acid residues that bind to peptidyl cis trans isomerase site resides of CypA(35). Therefore, these findings further confirmed that eCypA and CD147 were involved in regulating the secretion of inflammatory cytokines. The present study demonstrates that rMgPa could enhance the expression of CypA and CD147 in SV-HUC-1 cells. The findings of this study have suggested that CypA interacts with CD147, thus promoting the ERK phosphorylation and inflammatory cytokines secretion. However, many of the effects of MAPK on the inflammatory cytokines appear to be dependent on protein-protein interaction (36). rMgPa stimulation resulted in successful enhancement of the secretion of inflammatory cytokines after eCypA-CD147 interaction. In addition, the ERK-NF-κB pathway was evidently the downstream effect of eCypA-CD147 interaction during the inflammatory response process. Based on these findings of this study, it was evident that ERK phosphorylation resulting from eCypA-CD147 interaction was the upstream effect of MAPK signaling and that inhibition of this signaling axis significantly inhibited the expression of inflammatory cytokines. The results obtained in this study, therefore provided clear evidence that rMgPa induced eCypA played a central role in inducing ERK-mediated host cell responses during the rMgPa-mediated inflammatory response process.

Cytokines are crucial factors in inflammation and immunological diseases. MMPs are known to break down the ECM owing to the calcium and zinc required for their enzyme activity (37). Some MMPs have been well characterized and are known to have the ability to demote unequal substrates such as gelatin, collagen and elastin, which constitute the ECM. Excessive production of MMPs can cause massive loss of ECM which results in inflammation, arthritis, angiogenesis, cancer metastasis, and skin aging (38). Studies have also shown that the binding of eCypA to the membrane protein receptor CD147 could mediate the expression of related inflammatory cytokines and activated related enzymes (39). Similarly, this study verified that rMgPa-induced eCypA could stimulate SV-HUC-1 cells to express IL-1β, IL-6, and TNF-α. Interestingly, these results have validated that MMP-9, one of the matrix metalloproteinase enzymes (MMPs) from the MMP-s family, could be regulated by CD147.

Notably, this study only demonstrated the *in vitro* production of inflammatory cytokines via the CypA/CD147/ERK/NF-κB pathway by SV-HUC-1 cells stimulated by rMgPa-induced eCypA. Reports have shown that the intervention of CypA/CD147 signaling could probably provide a potential therapeutic and drug target for mice inflammatory periapical lesions progression (40), Glioblastoma (41), APE (42), HCMV (43), and so on. This study did not investigate the mechanism of interaction between CypA and CD147, the elucidation of which warrants further detailed studies. Previous studies suggested that inflammasomes play an important role in mycolasma infection, including *Mycoplasma pneumoniae* (44), *Mycoplasma salivarium* (45), *Mycoplasma hyorhinis* (46), and *Mycoplasma hyopneumoniae* (47). However, it is still unclear whether inflammatory cytokines secreted by MgPa-induced eCypA stimulated SV-HUC- cells are related to inflammasomes pathway. Therefore, we will further analyze the relationship between inflammatory cytokines production and the inflammasomes pathway in our next study. However, the findings of this study have suggested that rMgPa could induce eCypA secretion and thus evoke an inflammatory response via the CypA-CD147-ERK-NF-κB pathway in urethral epithelial cells, which enables better understanding of the pathogenesis and the pathogenic mechanism involved in *M. genitalium* infection. This study therefore lays the experimental foundation for further research aimed at elucidating the pathogenic mechanisms at play during infections caused by *M. genitalium*, which in turn could prove beneficial for the development of potential therapeutic and prophylactic candidates against this infection thus alleviating the challenges faced due to the chronic conditions resulting from this infection.

## Data Availability Statement

The raw data supporting the conclusions of this article will be made available by the authors, without undue reservation.

## Author Contributions

YZ and LL conceived and designed research. LL and DL conducted the experiments and analyzed the data. YL and KP contributed new reagents and analytical tools. LL wrote the manuscript. YZ revised the manuscript. All authors read and approved the manuscript.

## Conflict of Interest

The authors declare that the research was conducted in the absence of any commercial or financial relationships that could be construed as a potential conflict of interest.

## Abbreviations

CypA: Cyclophilin A
ECM: extracellular matrix
eCypA: extracellular Cyclophilin A
ERK: extracellular signal-regulated kinases
IL: interleukin
MMP: matrix metalloproteinases
MgPa: *Mycoplasma genitalium* protein of adhesion
NF: nuclear factor
qRT-PCR: quantitative reverse transcriptase polymerase chain reaction
rMaPa: recombinant *Mycoplasma genitalium* protein of adhesion
TNF: tumor necrosis factor.

## References

[1] Golden MR Workowski KA Bolan G. Developing a public health response to *Mycoplasma genitalium*. *J Infect Dis.* (2017) 216:S420–6. 10.1093/infdis/jix200 28838079 PMC5853686

[2] Vos T Barber RM Bell B Amelia BV Stan B Ian B Global, regional, and national incidence, prevalence, and years lived with disability for 301 acute and chronic diseases and injuries in 188 countries, 1990-2013: a systematic analysis for the Global Burden of Disease Study 2013. *Lancet.* (2015) 386:743–800. 10.1016/S0140-6736(15)60692-4 26063472 PMC4561509

[3] Sieving RE Gewirtz O’Brien JR Saftner MA Argo TA. Sexually transmitted diseases among us adolescents and young adults: patterns, clinical considerations, and prevention. *Nurs Clin North Am.* (2019) 54:207–25. 10.1016/j.cnur.2019.02.002 31027662

[4] Cohen CR Manhart LE Bukusi EA Brunham RC Holmes KK Sinei SK Association between *Mycoplasma genitalium* and acute endometritis. *Lancet.* (2002) 359:765–6. 11888591 10.1016/S0140-6736(02)07848-0

[5] Manhart LE Critchlow CW Holmes KK Dutro SM Eschenbach DA Stevens CE Mucopurulent cervicitis and *Mycoplasma genitalium*. *J Infect Dis.* (2003) 187:650–7. 12599082 10.1086/367992

[6] Mavedzenge SN Van Der Pol B Weiss HA Kwok C Mambo F Chipato T The association between *Mycoplasma genitalium* and HIV-1 acquisition in African women. *AIDS.* (2012) 26:617–24. 10.1097/QAD.0b013e32834ff690 22210630

[7] García B Merayo-Lloves J Martin C Alcalde I Quirós LM Vazquez F. Surface Proteoglycans as mediators in bacterial pathogens infections. *Front Microbiol.* (2016) 7:220. 10.3389/fmicb.2016.00220 26941735 PMC4764700

[8] Pich Oscar Q Burgos R Querol E Piñol J. P110 and P140 cytadherence-related proteins are negative effectors of terminal organelle duplication in *Mycoplasma genitalium*. *PLoS One.* (2009) 4:e7452. 10.1371/journal.pone.0007452 19829712 PMC2759538

[9] Dang B Li H Xu X Shen H Wang Y Gao A Cyclophilin A/Cluster of differentiation 147 interactions participate in early brain injury after subarachnoid hemorrhage in rats. *Crit Care Med.* (2015) 43:e369–81. 10.1097/CCM.0000000000001146 26132882

[10] McGowin CL Annan RS Quayle AJ Greene SJ Ma L Mancuso MM Persistent *Mycoplasma genitalium* infection of human endocervical epithelial cells elicits chronic inflammatory cytokine secretion. *Infect Immun.* (2012) 80:3842–9. 10.1128/IAI.00819-12 22907815 PMC3486055

[11] McGowin CL Radtke Andrea L Abraham K Martin DH Herbst-Kralovetz M. *Mycoplasma genitalium* infection activates cellular host defense and inflammation pathways in a 3-dimensional human endocervical epithelial cell model. *J Infect Dis.* (2013) 207:1857–68. 10.1093/infdis/jit101 23493725 PMC3654750

[12] Handschumacher RE Harding MW Rice J Drugge RJ Speicher DW. Cyclophilin: a specific cytosolic binding protein for cyclosporin A. *Science.* (1984) 226:544–7. 10.1126/science.6238408 6238408

[13] Wang P Heitman J. The cyclophilins. *Genome Biol.* (2005) 6:226. 10.1186/gb-2005-6-7-226 15998457 PMC1175980

[14] Soe NN Sowden M Baskaran P Kim Y Nigro P Smolock EM Acetylation of cyclophilin A is required for its secretion and vascular cell activation. *Cardiovasc Res.* (2014) 101:444–53. 24293519 10.1093/cvr/cvt268 PMC3928000

[15] Ramachandran S Venugopal A Sathisha K Reshmi G Charles S Divya G Proteomic profiling of high glucose primed monocytes identifies cyclophilin A as a potential secretory marker of inflammation in type 2 diabetes. *Proteomics.* (2012) 12:2808–21. 10.1002/pmic.201100586 22930659

[16] Deng X Dai P Yu M Chen L Zhu C You X Cyclophilin A is the potential receptor of the *Mycoplasma genitalium* adhesion protein. *Int J Med Microbiol.* (2018) 308:405–12. 29551599 10.1016/j.ijmm.2018.03.001

[17] Zhu D Wang Z Zhao JJ Calimeri T Meng J Hideshima T The Cyclophilin A-CD147 complex promotes the proliferation and homing of multiple myeloma cells. *Nat Med.* (2015) 21:572–80. 10.1038/nm.3867 26005854 PMC4567046

[18] Ghosh A Desai A Ravi V Narayanappa G Tyagi BK. Chikungunya virus interacts with heat shock cognate 70 protein to facilitate its entry into mosquito cell line. *Intervirology.* (2017) 60:247–62. 10.1159/000489308 29953983

[19] Zheng K Xu M Xiao Y Luo H Xie Y Yu J Immunogenicity and protective efficacy against *Treponema pallidum* in New Zealand rabbits immunized with plasmid DNA encoding flagellin. *Emerg Microbes Infect.* (2018) 7:177. 10.1038/s41426-018-0176-0 30405111 PMC6220273

[20] Opitz O Jacobs E. Adherence epitopes of *Mycoplasma genitalium* adhesin. *J Gen Microbiol.* (1992) 138:1785–90. 10.1099/00221287-138-9-1785 1383392

[21] Wang G Shen J Sun J Jiang Z Fan J Wang H Cyclophilin a maintains glioma-initiating cell stemness by regulating wnt/β-catenin signaling. *Clin Cancer Res.* (2017) 23:6640–9. 10.1158/1078-0432.CCR-17-0774 28790108

[22] Nigro P Pompilio G Capogrossi MC. Cyclophilin A: a key player for human disease. *Cell Death Dis.* (2013) 4:e888. 10.1038/cddis.2013.410 24176846 PMC3920964

[23] Jin ZG Melaragno MG Liao DF Yan C Haendeler J Suh YA Cyclophilin A is a secreted growth factor induced by oxidative stress. *Circ Res.* (2000) 87:789–96. 10.1161/01.res.87.9.789 11055983

[24] Su Z Lin M Zhang H Jiajie L Meiping W Hanlu L The Release of cyclophilin a from rapamycin-stimulated vascular smooth muscle cells mediated by myosin ii activation: involvement of apoptosis but not autophagy. *J Vasc Res.* (2020) 11:212–8. 10.1159/000506685 32526757

[25] Zhang M Schekman R. Cell biology. Unconventional secretion, unconventional solutions. *Science.* (2013) 340:559–61. 10.1126/science.1234740 23641104

[26] Dawar FU Tu J Khattak MN Mei J Lin L. Cyclophilin A: a key factor in virus replication and potential target for anti-viral therapy. *Curr Issues Mol Biol.* (2016) 21:1–20. 10.21775/cimb.021.001 27033630

[27] Liu L Li C Xiang J Dong W Cao Z. Over-expression and potential role of cyclophilin A in human periodontitis. *J Periodontal Res.* (2013) 48:615–22. 10.1111/jre.12047 23441725

[28] Kim SH Lessner SM Sakurai Y Galis ZS. Cyclophilin A as a novel biphasic mediator of endothelial activation and dysfunction. *Am J Pathol.* (2004) 164:1567–74. 10.1016/S0002-9440(10)63715-7 15111303 PMC1615642

[29] Wang CH Dai JY Wang L Jia JF Zheng ZH Ding J Expression of CD147 (EMMPRIN) on neutrophils in rheumatoid arthritis enhances chemotaxis, matrix metalloproteinase production and invasiveness of synoviocytes. *J Cell Mol Med.* (2011) 15:850–60. 10.1111/j.1582-4934.2010.01084.x 20455995 PMC3922672

[30] Wang L Wang CH Jia JF Ma XK Li Y Zhu HB Contribution of cyclophilin A to the regulation of inflammatory processes in rheumatoid arthritis. *J Clin Immunol.* (2010) 30:24–33. 10.1007/s10875-009-9329-1 19789967

[31] Damsker JM Bukrinsky MI Constant SL. Preferential chemotaxis of activated human CD4+ T cells by extracellular cyclophilin A. *J Leukoc Biol.* (2007) 82:613–8. 10.1189/jlb.0506317 17540735 PMC2846690

[32] Seizer P Ungern-Sternberg SN Schönberger T Borst O Münzer P Schmidt EM Extracellular cyclophilin A activates platelets via EMMPRIN (CD147) and PI3K/Akt signaling, which promotes platelet adhesion and thrombus formation in vitro and in vivo. *Arterioscler Thromb Vasc Biol.* (2015) 35:655–63. 10.1161/ATVBAHA.114.305112 25550208

[33] Tsai SF Hsieh CC Wu MJ Chen CH Lin TH Hsieh M. Novel findings of secreted cyclophilin A in diabetic nephropathy and its association with renal protection of dipeptidyl peptidase 4 inhibitor. *Clin Chim Acta.* (2016) 463:181–92. 10.1016/j.cca.2016.11.005 27823952

[34] Obchoei S Sawanyawisuth K Wongkham C Kasinrer W Yao Q Chen C Secreted cyclophilin A mediates G1/S phase transition of cholangiocarcinoma cells via CD147/ERK1/2 pathway. *Tumour Biol.* (2015) 36:849–59. 10.1007/s13277-014-2691-5 25296734

[35] Yurchenko V Zybarth G O’Connor M Dai WW Franchin G Hao T Active site residues of cyclophilin A are crucial for its signaling activity via CD147. *J Biol Chem.* (2002) 277:22959–65. 10.1074/jbc.M201593200 11943775

[36] Yi Y Liu Y Wu K Wu W Zhang W. The core genes involved in the promotion of depression in patients with ovarian cancer. *Oncol Lett.* (2019) 18:5995–6007. 10.3892/ol.2019.10934 31788074 PMC6865084

[37] Saif MW Li J Lamb L Kaley K Elligers K Jiang Z First-in-human phase II trial of the botanical formulation PHY906 with capecitabine as second-line therapy in patients with advanced pancreatic cancer. *Cancer Chemother Pharmacol.* (2014) 73:373–80. 10.1007/s00280-013-2359-7 24297682 PMC4123311

[38] Agere SA Akhtar N Watson JM Ahmed S. RANTES/ccl5 induces collagen degradation by activating MMP-1 and MMP-13 expression in human rheumatoid arthritis synovial fibroblasts. *Front Immunol.* (2017) 8:1341. 10.3389/fimmu.2017.01341 29093715 PMC5651228

[39] Chen W Zhao M Zhao S Lu Q Ni L Zou C Activation of the TXNIP/NLRP3 inflammasome pathway contributes to inflammation in diabetic retinopathy: a novel inhibitory effect of minocycline. *Inflamm Res.* (2017) 66:157–66. 10.1007/s00011-016-1002-6 27785530

[40] Flora GK Anderton RS Meloni BP Guillemin GJ Knuckey NW MacDougall G Microglia are both a source and target of extracellular cyclophilin A. *Heliyon.* (2019) 5:e02390. 10.1016/j.heliyon.2019.e02390 31517118 PMC6731207

[41] Wang YQ Zhang J Zhu LX Yu JJ Liu MW Zhu ST Positive correlation between activated CypA/CD147 signaling and MMP-9 expression in mice inflammatory periapical lesion. *Biomed Res Int.* (2019) 2019:8528719. 10.1155/2019/8528719 30949512 PMC6425416

[42] Xue C Sowden MP Berk BC. Extracellular and intracellular cyclophilin a, native and post-translationally modified, show diverse and specific pathological roles in diseases. *Arterioscler Thromb Vasc Biol.* (2018) 38:986–93. 10.1161/ATVBAHA.117.310661 29599134 PMC5920743

[43] Chen J Xia S Yang X Chen H Li F Liu F Human cytomegalovirus encoded miR-US25-1-5p attenuates CD147/EMMPRIN-mediated early antiviral response. *Viruses.* (2017) 9:365. 10.3390/v9120365 29194430 PMC5744140

[44] Segovia JA Chang TH Winter VT Coalson JJ Cagle MP Pandranki L NLRP3 is a critical regulator of inflammation and innate immune cell response during *Mycoplasma pneumoniae* infection. *Infect Immun.* (2018) 86:e00548-17. 10.1128/IAI.00548-17 29061706 PMC5736809

[45] Sugiyama M Saeki A Hasebe A Kamesaki R Yoshida Y Kitagawa Y Activation of inflammasomes in dendritic cells and macrophages by *Mycoplasma salivarium*. *Mol Oral Microbiol.* (2016) 31:259–69. 10.1111/omi.12117 26177301

[46] Xu Y Li H Chen W Yao X Xing Y Wang X *Mycoplasma hyorhinis* activates the NLRP3 inflammasome and promotes migration and invasion of gastric cancer cells. *PLoS One.* (2013) 8:e77955. 10.1371/journal.pone.0077955 24223129 PMC3819327

[47] Li B Du L Xu X Sun B Yu Z Feng Z Transcription analysis on response of porcine alveolar macrophages to co-infection of the highly pathogenic porcine reproductive and respiratory syndrome virus and *Mycoplasma hyopneumoniae*. *Virus Res.* (2015) 196:60–9. 10.1016/j.virusres.2014.11.006 25445346

