# Supplementary material for: Mycoplasma genitalium Protein of Adhesion Induces Inflammatory Cytokines via Cyclophilin A-CD147 Activating the ERK-NF-κB Pathway in Human Urothelial Cells
Source: Front Immunol. 2020 Sep 9;11:2052. doi: 10.3389/fimmu.2020.02052 (PMC7509115; doi:10.3389/fimmu.2020.02052)
Supplement: Supplementary file 1 [file Data_Sheet_1.pdf]

supplementary materials

Figure 1

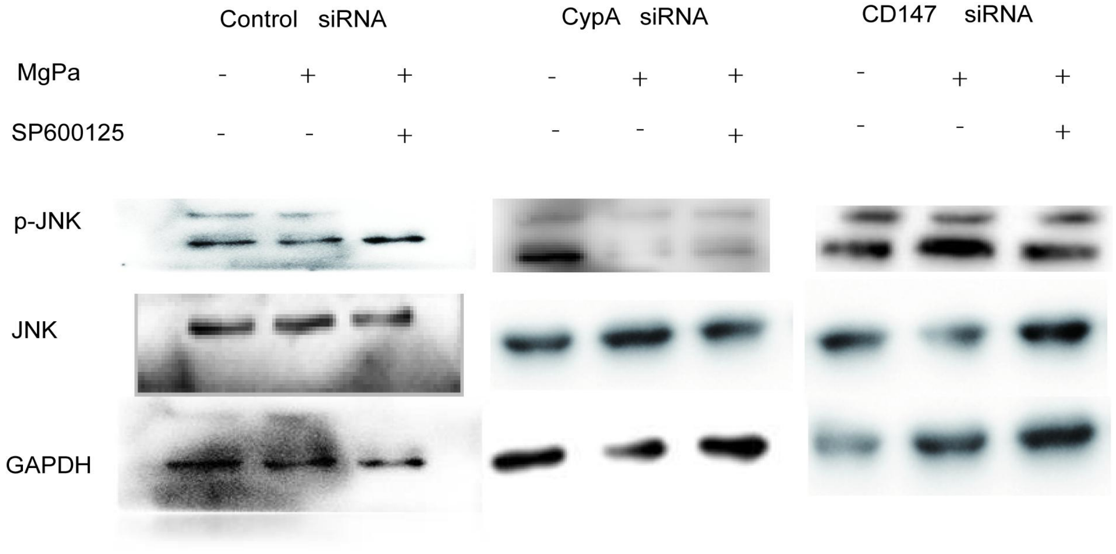

Figure 1 Cells were transfected with control siRNA or CypA siRNA or CD147 siRNA, as indicated at a final concentration of 20nM. After 48h, SV-HUC-1 cells were addressed with or without MgPa(20μg/mL). JNK and p-JNK protein levels were measured at 24h by Western blot. GAPDH was used as the control. SP600125 represent for the JNK inhibitor.

Figure 2

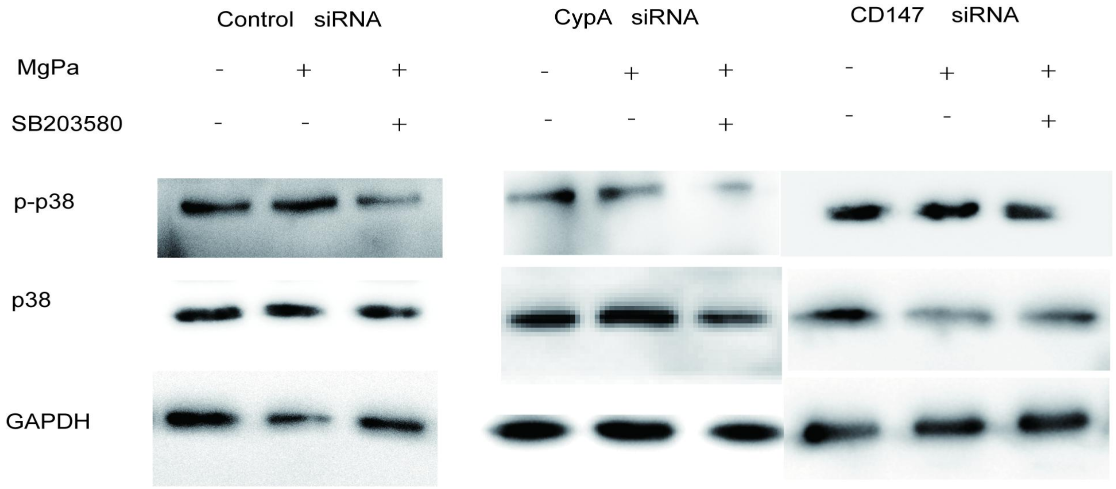

Figure 2 Cells were transfected with control siRNA or CypA siRNA or CD147 siRNA, as indicated at a final concentration of 20nM. After 48h, SV-HUC-1 cells were addressed with or without MgPa(20μg/mL). p38 and p-p38 protein levels were measured at 24h by Western blot. GAPDH was used as the control. SB203580 represent for the p38 inhibitor.
